# A Call for Bio-Inspired Technologies: Promises and Challenges for Ecosystem Service Replacement

**DOI:** 10.3390/biomimetics10090578

**Published:** 2025-09-02

**Authors:** Kristina Wanieck, M. Alex Smith, Elizabeth Porter, Jindong Zhang, Dave Dowhaniuk, Andria Jones, Dan Gillis, Mark Lipton, Marsha Hinds Myrie, Dawn Bazely, Marjan Eggermont, Mindi Summers, Christina Smylitopoulos, Claudia I. Rivera Cárdenas, Emily Wolf, Peggy Karpouzou, Nikoleta Zampaki, Heather Clitheroe, Adam Davies, Anibal H. Castillo, Michael Helms, Karina Benessaiah, Shoshanah Jacobs

**Affiliations:** 1 Biomimetics and Innovation, Deggendorf Institute of Technology, Faculty of Applied Informatics, Grafenauer Str. 22, 94078 Freyung, Germany; kristina.wanieck@th-deg.de (K.W.); jindong.zhang@th-deg.de (J.Z.); emily.wolf@stud.th-deg.de (E.W.); 2 Department of Integrative Biology, College of Biological Sciences, University of Guelph, 50 Stone Road East, Guelph, ON N1G 2W1, Canada; salex@uoguelph.ca (M.A.S.); eporte02@uoguelph.ca (E.P.); acastill@uoguelph.ca (A.H.C.); 3 Department of Biology, Laboratory of Functional Morphology (FunMorph), University of Antwerp, 2000 Antwerp, Belgium; 4 School of Theatre, English and Creative Writing, College of Arts, University of Guelph, 87 Trent Ln, Guelph, ON N1G 1Y4, Canada; ddowhani@uoguelph.ca (D.D.); liptonm@uoguelph.ca (M.L.); 5 Department of Population Medicine, Ontario Veterinary College, University of Guelph, 50 Stone Road East, Guelph, ON N1H 2P9, Canada; aqjones@uoguelph.ca; 6 School of Computer Science, College of Engineering and Physical Sciences, University of Guelph, 50 Stone Road East, Guelph, ON N1G 2W1, Canada; dgillis@uoguelph.ca; 7 Department of Political Science, College of Social & Applied Human Sciences, University of Guelph, 50 Stone Road East, Guelph, ON N1G 2W1, Canada; mhindsmy@uoguelph.ca; 8 MacMillan Center for International & Area Studies, Yale University, Henry R. Luce Hall, 34 Hillhouse Avenue, New Haven, CT 06511, USA; dawn.bazely@yale.edu; 9 Department of Mechanical and Manufacturing Engineering, Schulich School of Engineering, University of Calgary, Calgary, AB T2N 1N4, Canada; meggermo@ucalgary.ca; 10 Department of Biological Sciences, University of Calgary, 507 Campus Dr NW, Calgary, AB T2N 4V8, Canada; mindi.summers@ucalgary.ca; 11 School of Fine Art and Music, College of Arts, University of Guelph, 50 Stone Road East, Guelph, ON N1G 1V7, Canada; csmylito@uoguelph.ca; 12 Instituto de Ciencias de la Atmósfera y Cambio Climático, Universidad Nacional Autonoma de Mexico, Circuito Exterior s/n, C.U., Coyoacán, Mexico City 04510, Mexico; claudia.rivera@ciencias.unam.mx; 13 Faculty of Philology, School of Philosophy, National and Kapodistrian University of Athens, Panepistimiopolis, Ilissia, 15784 Athens, Greece; pkarpouzou@phil.uoa.gr (P.K.); nikzamp@phil.uoa.gr (N.Z.); 14 Department of English, Faculty of Arts, University of Calgary, 2500 University Dr NW, Calgary, AB T2N 1N4, Canada; hjclithe@ucalgary.ca; 15 Department of Family Relations and Applied Nutrition, College of Social and Applied Human Sciences, University of Guelph, 88 McGilvray St, Guelph, ON N1G 2W1, Canada; adam.davies@uoguelph.ca; 16 School of Mechanical Engineering, Georgia Institute of Technology, 801 Ferst Dr NW, Atlanta, GA 30318, USA; michael.helms@me.gatech.edu; 17 Department of Geography, Environment and Geomatics, College of Social and Applied Human Sciences, University of Guelph, 50 Stone Road East, Guelph, ON N1G 2W1, Canada; kbenessa@uoguelph.ca

**Keywords:** biomimetics, biologically inspired design, climate change, soil biodiversity

## Abstract

Ecosystem services are crucial for animals, plants, the planet, and human well-being. Decreasing biodiversity and environmental destruction of ecosystems will have severe consequences. Designing technologies that could support, enhance, or even replace ecosystem services is a complex task that the Manufactured Ecosystems Project team considers to be only achievable with transdisciplinarity, as it unlocks new directions for designing research and development systems. One of these directions in the project is bio-inspiration, learning from natural systems as the foundation for manufacturing ecosystem services. Using soil formation as a case study, text-mining of existing scientific literature reveals a critical gap: fewer than 1% of studies in biomimetics address soil formation technological replacement, despite the rapid global decline in natural soil formation processes. The team sketches scenarios of ecosystem collapse, identifying how bio-inspired solutions for equitable and sustainable innovation can contribute to climate adaptation. The short communication opens the discussion for collaboration and aims to initiate future research.

## 1. Planetary Boundaries and Ecosystem Services

The future of human well-being is linked to well-functioning Ecosystem Services (ESs). ESs provide resources for basic survival, like food, fresh air, and clean water; belonging needs, like healthy social relations; and esteem needs, like an adequate livelihood and cultural freedom [1]. The United Nations’ Sustainable Development Goals, which “ask humanity to act,” are implicated in healthy ES functioning [2].

At present, anthropogenic activities surpass six of nine planetary boundaries [3]. The destruction of natural ecosystems links biodiversity loss to the rate of mass extinction [4]. With biodiversity loss comes the reduced functioning of ESs. Common sense mistakenly views ESs as only beneficial for human well-being—this is a critical misnomer. ESs are crucial. If ESs collapse, human well-being will be permanently impacted, and the consequences will be severe. As one example, we are all witness to the consequences of declining diversity of wild pollinators, which directly reduces food production [5].

The Manufactured Ecosystems (MEco) project asks critical questions: “Can ES be entirely replaced? What is needed to build technological proxies for ES? To what extent can we leverage technology to replace ecosystem services? Which biological systems could serve as inspiration for new technologies?” Like Fitter’s work on supporting or enhancing ESs [6], the MEco project envisions plausible disaster scenarios that consider how to mitigate likely climate crises to prevent total collapse as we try to adapt. 

**Figure 1:**
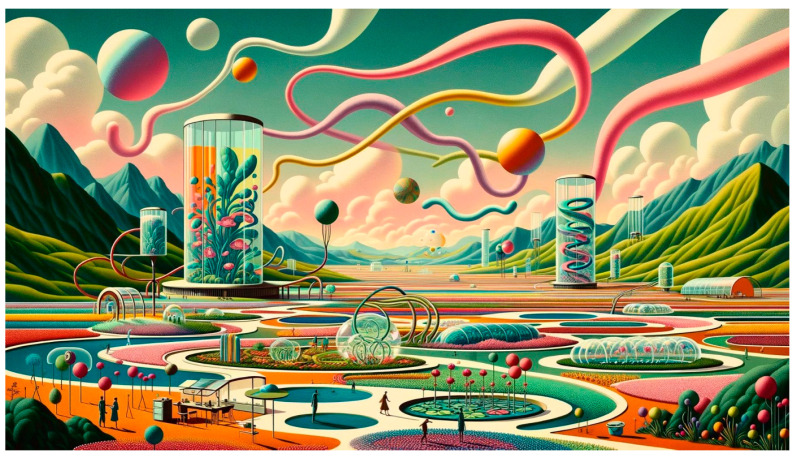
An envisioned Manufactured Ecosystem. The image shows an AI-generated scenario of a total replacement of ESs by technology as part of the science fiction work in the MEco project. Image generation: Nicolas Leo Moreira and OpenAI.

Though we understand that a full technological replacement is unscalable, we envision a scenario of a total replacement of natural systems by technology to imagine if humans would want to live in an environment, where for example, tiny robots take over the work of pollination [7], laboratories contain the power of all food production, and virtual, augmented reality is the only means for social connection [8]. Figure 1 shows an AI-generated picture of such an envisioned MEco.

The answer to this question for the authors of this article, of course, is no. Yet, if we are to avoid a post-climate apocalyptic reality, researchers from various disciplines, artists, community knowledge holders, creative thinkers, and strategic problem solvers are called on for innovations and technologies to help the world prepare and mitigate risks. What is needed now are new (e.g., to research) tools and languages for understanding the natural world, new strategies for preventative and adaptive action, and changes that will support currently endangered ESs. We cannot design our way out of this global crisis using the approaches that we used to cause it. Those approaches were partly based on exploitative systems that devalued nature and living with nature.

The transdisciplinary MEco team investigates ecosystems to learn from their biodiversity: Bio-inspiration—or nature-based solutions—are a means for developing technologies “conducive to life” [9], also considering the ethical implications of bio-inspired developments [10], an especially important aspect in the context of enhancing or supporting crucial ESs as they provide the fundamental requirements for life for any living being. 

The goal of the project team is to co-create with biodiversity; living organisms are not used in any solution. Instead, the team looks to nature to learn. Through careful observation and learning from living organisms, they can abstract unique principles that can be used to develop new technologies.

## 2. Soil as an Example—A Provisioning ES

Soil is the most biologically rich habitat: “A teaspoon of soil contains more living beings than humans on Earth” [11] and is of inestimable value. In the European Union alone, more than 60 percent of soils are considered damaged [12,13], reducing their ES functionality (e.g., degraded soils in Bavaria, Germany [13]). Healthy soil stores more gas carbon dioxide (CO_2_) than forests and is second only to oceans [14]. It stores water and buffers the effects of the climate crisis, including drought, heavy rainfall, and floods [15].

**Figure 2:**
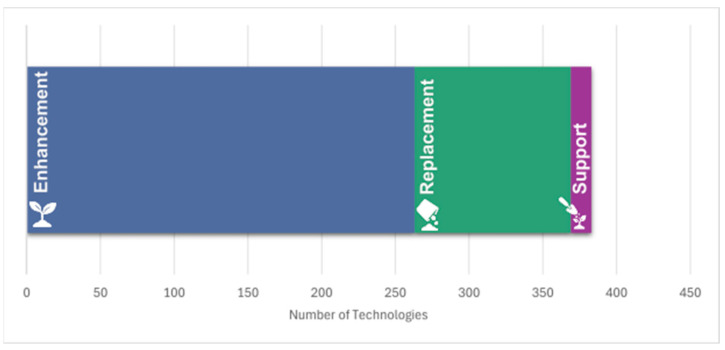
Technologies described in the research literature that Enhance, Replace, or Support soil formation ecosystem services. The search was conducted using a Large Language Model (LLM) and the query “biomim* (soil) OR bio*inspir* (soil).

Soil is a co-working space of more than ten different phylogenetic branches of biodiversity that likely includes tens of thousands of species [16], each with a role in providing healthy soil. Yet, in a global search of technologies related to the functions of soil as an ES provider, only 84 articles in Web of Science (search limited to English language) were retrieved that dealt with the topic of “ecosystem service technologies AND soil”, primarily focusing on phytoremediation (see below) and water purification. Of these articles, only 24 could be of value for identifying supporting or enhancing technologies. No articles described a bio-inspired approach that begins by learning from natural systems. A more differentiated picture emerges when we analyze the broader bio-inspiration corpus. Text-mining using a commercial large language model (LLM) and the query “biomim* (soil) OR bio*inspir* (soil)” revealed 383 publications: 263 are linked to enhancement technologies, 106 aim at replacement, and 14 focus on support (Figure 2). What we find is that the difference between these numbers and the publications yielded using “ecosystem service technologies AND soil” searches of the literature indicates that ecosystem-service research is not directly linked to bio-inspired approaches. And bio-inspiration studies have concentrated on technological solutions without necessarily recognizing their potential for system-level impact. Further analysis will reveal existing and non-existing research topics in the context, and it will indicate which biological models are described in those subsets of literature. Given the current state of the (English) scientific literature, we do not come close to being able to enhance, replace, or support the services provided by healthy soils. By combining learning from both the existing literature and soils themselves, we can gain insights into biological functions and processes. 

With the rapid advancement of the climate crisis, we can leverage our collective research goals towards climate adaptation technologies. This knowledge can guide the creation of new, innovative technologies to enhance, replace, or support natural ESs. 

## 3. Biodiversity as a Model

The biomimetic approach does not use the biological systems themselves but abstracts the underlying principles of a function observed in the natural system [17]; it understands biological systems as ‘field-tested technology’, with solutions to ubiquitous problems. 

**Figure 3:**
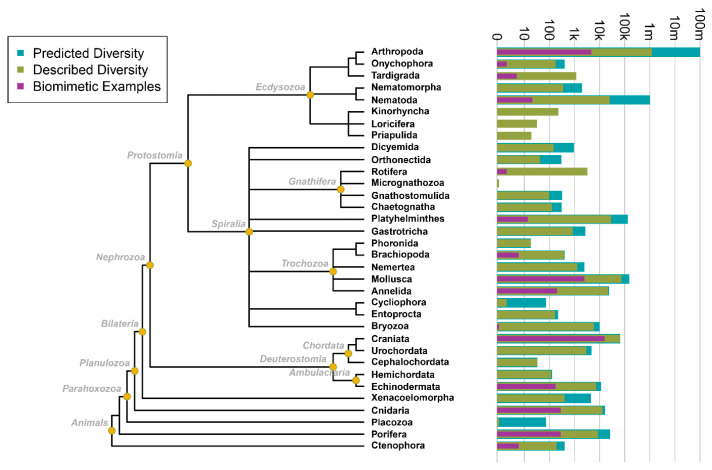
The phylogenetic breadth of biological systems used for bio-inspired technologies. This phylogenetic tree has been developed, mapping predicted (blue) and described (green) diversity (phylogeny adapted from Dunn et al. [22]—a hypothesis of animal phylogeny, compiled across multiple studies and methodologies) against their usage frequency in bio-inspirations (purple) within the animal kingdom. The “Biomimetic Examples” (purple) illustrates the total frequency at which biological systems have been used as inspiration, based on the number of biological systems used for the inspiration of documented 379 products [20] combined with data from an extensive literature analysis [23], i.e., these frequency counts reflect the number of times models within each phylum were cited, rather than the number of distinct taxonomic groups utilized.

Approximately 2.3 million animal species have been named—yet total global biodiversity may include 100 s of millions of species [18]. Similarly, most organisms used as models for technological developments come only from well-known species. This indicates that the diversity used in bio-inspired applications is limited and biased [19,20] and calls upon us to recognize the importance of transdisciplinary research support and collaboration. Figure 3 shows the breadth of biological organisms that have been or could be used for the development of bio-inspired technologies. Most biodiversity is yet to be used for inspiration, as bio-inspired research has only scratched the surface of possibilities [21]. 

These unnamed contributors to global biodiversity represent an immense and unrealized potential for inspiring new technologies. We cannot model new technologies based on taxa about whose pathways through this world we are unaware. For example, most of these unknown organisms likely experience the world through smell and taste, not vision and sound. These unknown unknowns, the lineages and species that we have not yet looked at or known to look at, likely hold the key to many efficient bio-inspired strategies and services that human society needs. 

In contrast to biomimetics, some of the existing ES enhancement or replacement technologies make use of the biological models themselves in application and bio-utilization. One example is phytoremediation (mentioned above), where plants and associated soil microbes are used to reduce the concentrations or toxic effects of contaminants in the environment by up-taking them. Phytoremediation is widely accepted as a cost-effective environmental restoration technology [24]. Using a biomimetic approach, we would begin by learning from the services provided by the organisms in soil (e.g., nutrient cycling, water purification), abstracting the functional principles and mechanisms to design technologies to restore or support ES functionality. 

While Fitter, 2013 [6] focused on bio-technological solutions, we shift to bio-inspired and especially biomimetic solutions as a means of addressing ES loss. Our work reveals that biomimetic solutions are underrepresented in ES-related technology. Biomimetics offers access to a realm of existing natural solutions that could operate in partnership with natural ES functioning, and many of the challenges of conducting biomimetic research specifically are well-known, and so are the approaches to overcome them [25,26,27,28,29]. But applying biomimetic technologies to enhance/replace/support ESs will require new ways of envisioning the traditional disciplines and systems of research practice, and a paradigm shift in how [30] and why we innovate. 

## 4. Transdisciplinary Teamwork

To consider the holistic requirements in biomimetic developments, it is necessary to include philosophy, art, industry, business, politics, and community institutions [10]. In the MEco project, a team of 21 researchers from six countries and 12 very different academic fields work together, supported by 20 early-career researchers (undergraduate and graduate students), artists, and science-fiction authors. This unique transdisciplinary cooperation with a bio-inspiration mindset is the next step in recognizing the engineering potential of bio-inspiration and biomimetics. The call for greater support of transdisciplinary research has gone unanswered [31,32], and we are fast approaching the point beyond which only transdisciplinary solutions will be required. MEco is committed to transdisciplinarity for its usefulness in challenging knowledge silos and creating space to ensure the populations who stand to be most affected by ecosystem failure are also those that are included in the conversations and ideations about solutions, e.g., reference [31]—populations often excluded.

Considering the rapid pace at which our global soil security is declining [33], we require international transdisciplinary endeavors to adopt new design approaches to access existing knowledge systems about the natural world. New AI-based tools for accessing that knowledge [34,35] can speed up developments that we miss in scientific literature, but, ultimately, new ways of human-based collaboration will be the lasting approach. A drastic overhaul of national research funding systems, prioritizing transdisciplinary exploration and innovation, will go a long way to developing the solutions we need to adapt to our collapsing climate.

## Data Availability

Data are available upon request.

## Abbreviations

The following abbreviations are used in this manuscript:

ES	Ecosystem Services
LLM	Large Language Model
MEco	Manufactured Ecosystems
